# Comparison of Ultrasound Versus Ultrasound with Nerve Stimulator-Guided Infraclavicular Block Anesthesia Methods in Pediatric Patients

**DOI:** 10.3390/medicina61060985

**Published:** 2025-05-27

**Authors:** Abdulhakim Şengel, Evren Büyükfirat, Selçuk Seçilmiş, Nuray Altay, Ahmet Atlas, Abdullah Şengül

**Affiliations:** 1 Department of Anesthesiology and Reanimation, Faculty of Medicine, Harran University, 63040 Şanlıurfa, Turkey; evrenbf@gmail.com (E.B.); nurayaltay@ymail.com (N.A.); ahmetatlas@harran.edu.tr (A.A.); abdullahsengul342@gmail.com (A.Ş.); 2 Department of Anesthesiology and Reanimation, Kahta State Hospital, 02400 Adiyaman, Turkey; sleon_02@hotmail.com

**Keywords:** analgesia, anesthesia, brachial plexus block, nerve block, upper extremity, patients, pediatrics

## Abstract

*Background and objectives*: Brachial plexus block is one of the most effective anesthesia and analgesia methods for upper extremity surgeries across different age groups. However, the number of studies on this block in children is insufficient. The aim of this study was to retrospectively analyze and discuss the efficacy and safety of ultrasound (US)- and Ultrasound with nerve stimulator (US + NS)-guided infraclavicular brachial plexus block (ICB) in pediatric patients. *Materials and Method*: In this study, we retrospectively analyzed the data of 240 pediatric patients admitted to our clinic between October 2020 and April 2023, 120 of whom underwent US-guided ICB and 120 who underwent US + NS-guided ICB. *Results*: Demographic data of both groups who underwent US and US + NS-guided ICB were similar. The mean procedure time was 6.1 ± 0.8 min for the US group and 8.31 ± 0.82 min for the US + NS group (*p* < 0.001). The mean operative time was 62.4 ± 11.3 min in the US group and 62.4 ± 9.5 min in the US + NS group (*p* = 0.73). Intraoperative and postoperative opioid and additional analgesia use and pain scores at 1, 3, 6, 9, 12, 15, and 24 h were recorded in both groups. The mean duration of the motor block (MBD) was 6.20 ± 0.95 h in the US group and 6.29 ± 0.88 h in the US + NS group (*p* = 0.46). The mean duration of sensory block (SBD) was 9.38 ± 2.13 h in the US group and 9.53 ± 2.05 h in the US + NS group (*p* = 0.38). *Conclusions*: In pediatric patients, US and US + NS-guided ICB applications are effective and safe in ease of application, prolonged analgesia, and low complication rates. In skilled hands, US-guided ICB can be as effective as US + NS-guided ICB. Further prospective studies with more significant patient populations are needed to validate these findings.

## 1. Introduction

Upper extremity surgery represents the majority of orthopedic procedures in pediatric patients [1]. However, compared to adults, regional anesthesia (RA) studies in the pediatric age group are limited [2]. Interscalene, supraclavicular, infraclavicular, and axillary approaches for brachial plexus block have been described in upper extremity surgery. However, axillary block is widely used in pediatric patients because of its safety and ease of application instead of interscalene and supraclavicular approaches due to possible risks such as cervical and phrenic nerve involvement, arterial puncture, Horner’s syndrome, and pneumothorax [3]. On the other hand, disadvantages such as the need for multiple approaches and inadequate analgesia have also been reported [4]. Infraclavicular nerve block (ICB) has also been described in pediatric patients using both nerve stimulator (NS) and ultrasound (US) guidance [4]. This study aimed to compare the effects of two different infraclavicular brachial plexus block (ICB) methods, performed under ultrasound (US) guidance alone and combined ultrasound and nerve stimulator (US + NS) guidance, on the procedure time, motor block duration (MBD), sensory block duration (SBD), and the quality of analgesia over 24 h postoperatively in pediatric patients undergoing arm, forearm, and hand surgery.

## 2. Material and Method

This study was designed as a clinical study resulting from analyzing retrospectively collected data. It was conducted retrospectively after the approval of the Harran University Clinical Research Ethics Committee (5 June 2023 and 23/10/03 decision).

As this was a retrospective observational study, patients were not randomly allocated but grouped based on the anesthesia technique recorded in clinical files. No matching analysis was performed; however, the groups were similar in terms of age, gender, ASA classification, and BMI.

### 2.1. Study Population

Between October 2020 and April 2023, 240 patients between the ages of 2 and 15 years, ASA I-II group according to the American Society of Anesthesiologists (ASA) classification, who were scheduled to undergo arm, forearm, and hand surgery, were enrolled in the study. Patients with ASA III–IV–V group, contraindications to a peripheral nerve block, immune function and severe inflammatory response, cognitive and nervous system disorders, known anesthetic allergy, paresthesia, motor nerve damage, contralateral hemidiaphragm dysfunction, phrenic nerve injury, and whose parents did not accept the block procedure were excluded.

After the patients were admitted to the preoperative unit, 15 mL/kg of fluid was started, and the patients were premedicated with midazolam (Zolamide 5 mg/mL) i.v. at 0.04 mg/kg. Ketamine i.v. at a dose of 1 mg/kg was additionally administered to patients who could not achieve adequate sedation with midazolam.

Two senior anesthesiologists with over 5 years of experience in pediatric regional anesthesia performed all blocks to ensure consistency and minimize operator-related variability. Ultrasound-guided blocks were performed using a high-frequency (10–18 MHz) linear probe (Esaote My Lab 30 Gold, Esaote North America Inc., Indianapolis, IN, USA). This frequency allowed optimal fascicle visualization, which is critical for block success in pediatric patients [5].

In patients undergoing ICB with US guidance alone, the long-axis method was used to visualize the axillary artery and the surrounding neural structures (lateral, medial, and posterior cords). The block needle was then directed to administer the local anesthetic (LA) solution around all three cords. After reaching the desired areas, a negative aspiration test was performed (repeated after each 3 mL LA injection) and 2% lidocaine (Lidon 100 mg/5 mL On Farma) at 2 mg/kg and 0. 5% bupivacaine (Buvasin 5 mg/mL, Vem İlaç, İstanbul, Turkey) at 1 mg/kg and 0.9% isotonic sodium chloride diluted mixture up to the calculated total volume of LA solution were prepared and injected into the relevant areas.

In the patient group undergoing block with US + NS guidance, the position of the block needle was determined under ultrasound visualization and NS guidance. The needle was advanced until no reflex response was observed at 0.2 mA stimulation. Still, a reflex response was elicited at 0.3 mA depending on the stimulated cord (forearm pronation for lateral cord stimulation, wrist extension for posterior cord stimulation, and wrist flexion for medial cord stimulation). After confirming the correct positioning, half of the prepared LA mixture was administered to the posterior cord. In contrast, the remaining half was equally distributed between the lateral and medial cords to complete the block procedure.

The success of the block was recorded 15 min after the block procedure in both groups after evaluation of the Bromage scale, cold sensory loss test, and pinprick test in the extremity where the block was performed for control. If the block was successful and there was no significant hemodynamic change, the patient was transferred to the operating room, and surgery was started approximately 30 min after the block procedure.

According to ASA recommendations, standard monitoring (ECG, tachycardia, SpO_2_) was performed on patients brought to the operating table. Oxygen was administered with an oxygen mask at 3–4 L/min until the end of surgery. Patients’ hemodynamic parameters were recorded throughout the operation. Intraoperative opioid administration was recorded. Possible complications (pneumothorax, neurological damage, hematoma, and Horner’s syndrome) were recorded.

After the brachial plexus block, the time to visualize finger abduction was defined as MBD. The time from the brachial plexus block to the first rescue analgesic administration was defined and recorded as SBD. After admission to the recovery room and during ward follow-up, the pain was assessed using the Face, Leg Movement, Action, Moaning, Consoling (FLACC) scale for patients aged 2–7 years and the Visual Analog Scale (VAS) for patients aged 8–15 years. Paracetamol (Parolivflacon, Atabay Kimya, Istanbul, Turkey) 10 mg/kg i.v. was administered every six hours postoperatively. In patients with a pain score of 4 or higher according to FLACC or VAS score, 7.5 mg/kg oral ibuprofen (Ibufen 100 mg/5 mL, AbbottLabs, Istanbul, Turkey) was administered as a rescue analgesic. Due to institutional protocol, all pediatric patients were observed for at least 24 h after regional anesthesia. Although scheduled, not all patients attended follow-up for an outpatient clinic visit to assess for possible complications.

### 2.2. Sample Size

The sample size for this study was determined retrospectively based on the postoperative sensory block duration (SBD) and motor block duration (MBD) as primary outcome variables. According to previous similar studies, the mean sensory block duration was reported as 9.0 ± 2.0 h, and the mean motor block duration was reported as 6.2 ± 1.0 h. An expected mean difference of 0.5 h for SBD and 0.3 h for MBD between the groups (US and US + NS) was assumed.

Post hoc power analysis was conducted. Power analysis was performed using these values with a two-tailed significance level (α) of 0.05 and a statistical power (1 − β) of 80%. The effect sizes (Cohen’s d) were calculated as 0.25 for SBD and 0.30 for MBD. Based on these effect sizes, the minimum required sample size was 88 participants per group for SBD and 74 participants per group for MBD.

This study included 120 participants in each group (US and US + NS), exceeding the minimum required sample sizes. The larger sample size ensured sufficient statistical power to detect significant differences in sensory and motor block durations, providing robust and reliable results. The sample size calculations were performed using the statistical software G*Power (version 3.1.9.2; Franz Faul and Edgar Erdfelder, Trier, Germany).

### 2.3. Statistical Analysis

SPNS 22.0 for Windows was used for statistical analysis. Descriptive statistics were reported as numbers and percentages for categorical variables and mean, standard deviation, minimum, maximum, and median for numerical variables. Numerical variables were compared using two independent Mann–Whitney U tests because the normal distribution condition was not met. Group proportions were compared using the chi-squared test. The alpha level of statistical significance was accepted as *p* < 0.05.

## 3. Results

All brachial plexus blocks were successfully performed with the sedation regimen described in the materials and methods section. No adverse events, including pneumothorax, nerve injury, hematoma, or Horner’s syndrome, were observed or recorded in either group.

No statistically significant difference was observed in the demographic characteristics of the groups (Table 1).

The procedure time of the US + NS group was statistically significantly higher than that of the US group (*p* < 0.001) (Table 2).

**Figure 1:**
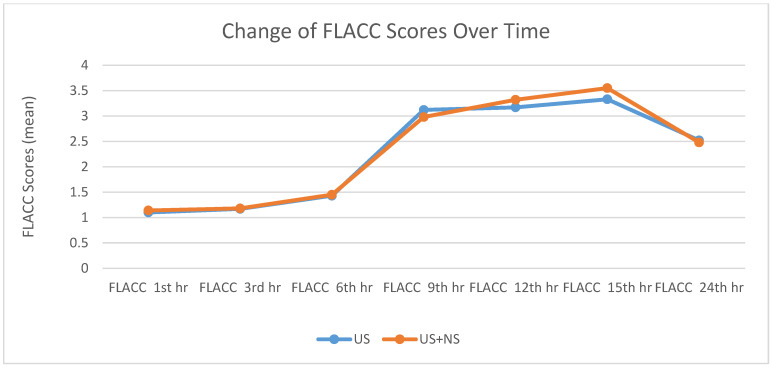
Change in mean FLACC scores over time. FLACC: Face, Leg Movement, Wailing, Action scale; US: ultrasound; US + NS: ultrasound with nerve stimulator.

**Figure 2:**
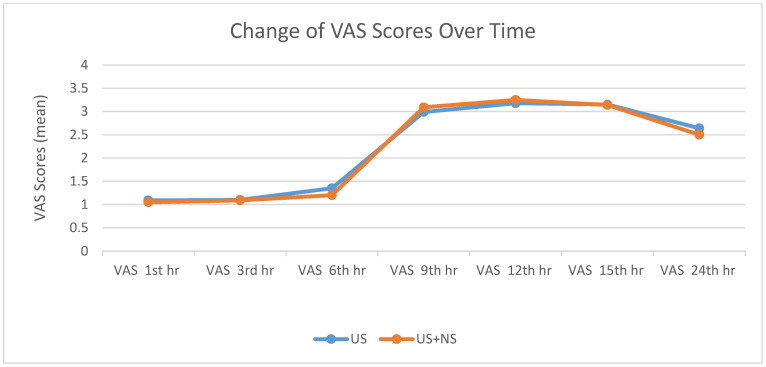
Change in mean VAS scores over time. VAS: Visual Analog Scale; US: ultrasound; US + NS: ultrasound with nerve stimulator.

There was no statistically significant difference in the pain sensations of the groups (Table 3, Table 4 and Table 5, Figure 1 and Figure 2).

There was no statistically significant difference between the groups at the time of the first postoperative analgesic requirement, and the total amount of analgesic used was MBD and SBD (Table 6).

## 4. Discussion

The data obtained in this study aimed to evaluate the experience of using RA as a primary anesthesia method in pediatric patients. It was determined that the US-guided block was at least as effective and reliable as the US + NS-guided block in pediatric patients.

Due to technology development, the range of applications in the US has expanded, and their cost has decreased, contrary to what was previously stated. This has made using the US in regional anesthesia techniques inevitable in pediatric age groups and adults [6]. The anesthesiologist’s experience is a key factor influencing the success and safety of regional blocks. In this study, the high success rate and absence of complications may be attributable to the fact that all procedures were performed under ultrasound guidance by experienced senior anesthesiologists.

Due to the widespread use of the US and the increase in the number of experienced and qualified regional anesthesiologists, ICB can easily be performed successfully in shorter times. Looking at the literature, longer times are reported in this sense. In the study by Altinay et al., this time was 12.9 ± 2.8 min [7,8]. In this study, these times were 6.1 ± 0.8 min in the US group and 8.31 ± 0.82 min in the US + NS group. When the block procedure times were compared between groups, there was a clinically minimal difference (*p* < 0.001) lower in the US group than in the US + NS group.

This study is similar to the previous study by İnce et al. on SBD and MBD [9]. Since the duration of SBD and MBD was recorded based on the end of the block procedure, it was found that there was no significant difference between the two groups in this sense (*p* = 0.382 for SBD and *p* = 0.460 for MBD). However, when the block procedure time was taken as the basis, a significant difference was found in favor of the US group (*p* < 0.001). In this sense, this study is similar to the literature, which states that ultrasound imaging shortens the duration of pain sensation in children by providing shorter sensory onset times compared to NS guidance [4].

In previous studies, many pain scores have been used in the intraoperative and postoperative process in the pediatric age group [9]. This study used the FLACC score for patients aged 0–7 years and the VAS score for patients aged 8–15 years. In both scores, scores of 4 and above were considered the threshold for additional analgesia.

Correctly performed infraclavicular block provides hemodynamic stability, reduces catecholamine production and metabolic stress response to surgery, decreases the incidence of postoperative respiratory complications, and promotes rapid return of bowel function and nutrition [10]. In this study, hemodynamic instability and postoperative respiratory complications were not observed in either group.

Other studies in children have shown that RA significantly reduces postoperative opioid use [11,12,13,14]. In this study, no intraoperative or postoperative opioid use was required in the first 24 h (Table 6).

A prospective multicenter cohort meta-analysis study that reviewed more than 100,000 nerve blocks reported that RA in children was at least as safe as in adults and showed that nerve and stimulator techniques changed with the development of US technology. However, most nerve blocks were performed under general anesthesia [15]. As a result, it is recommended that regional anesthesia in children be performed under general anesthesia or deep sedation [16]. Pediatric patients in the younger age group may be noncompliant during the block phase, and the compliance rate for the simple procedure increases with age [17]. In this study, the block procedure was performed under deep sedation (ketamine + midazolam) in younger patients and under light sedation (midazolam) in older patients with compliance problems. There was no significant difference in the administration of sedation between the two groups (*p* = 0.784) (Table 2). As the block procedure was successfully performed under sedation in both groups, general anesthesia was not required in any patient.

Due to physiological, anatomical, and pharmacodynamic changes in the pediatric age group, LAs are used at lower concentrations compared to adults, thus reducing the risk of systemic toxicity [17]. Again, in a US-guided block procedure, it may be helpful to administer a lower volume of LA compared to the block procedure performed with NS or classical methods [18]. In the US-guided block procedure, the target nerves are directly visualized, and the distribution of LA is monitored, preventing nerve damage from the block procedure and unwanted distribution of LA [7]. Therefore, US-guided nerve block increases the success rate of the block, prolongs the block time, and reduces the number of needle punctures [19]. In light of these data, the block procedures performed in both groups in this study were performed under low dose, low volume, and low LA concentration. In addition, no intravascular or intrafascicular injection was detected. Therefore, in this study, it was determined that the US facilitates the blocking process and improves the quality of the procedure, which supports the studies in the literature.

The use of multimodal analgesics, a model in which multiple agents are used together for analgesia and anesthesia, has been shown to further reduce the risk of side effects compared to single-drug therapy [20,21]. Multimodal analgesia, which is an alternative to opioid monotherapy in the treatment of acute pain, is a non-opioid-based approach with the addition of adjunctive opioids as needed. When RA is part of multimodal analgesia, patient recovery is improved, discharge times are shortened, and a rapid return to daily activities is ensured [22]. There are very few studies on the dose of LA to be used in the block procedure in pediatric patients. Ince et al. administered a 1:1 mixture of 0.5% bupivacaine and 2% lidocaine in a 0.5 mL/kg volume. In his study, Ponde reported that 2% lidocaine with adrenaline can be used up to 7 mg/kg, and in another study, Fettiplace reported that 0.5% bupivacaine can be used up to 3 mg/kg in patients aged 2–15 years [9,17,23]. In the blocks performed in this study, 0.5% bupivacaine was prepared as 1 mg/kg, and 2% lidocaine was prepared as 1.5 mg/kg with 0.9% NaCl in a 1:1 ratio and administered to the patients in a volume of 0.5 mL/kg. In this way, the rapid effect of the block was ensured, and high single-agent doses and other side effects, especially systemic LA poisoning, were prevented. In addition, the use of opioids for the treatment of acute postoperative pain was reduced, and a faster return to daily activities.

### Limitations

One limitation of our study is the 24 h follow-up period. Although outpatient visits were scheduled, many patients did not attend, limiting our ability to evaluate long-term outcomes or delayed complications.

## 5. Conclusions

This study is one of the few to investigate the effects of US, US + NS-guided ICB on procedure time, MBD, SBD, and quality of analgesia during the postoperative 24 h for RA in pediatric patients. Based on our cohort, US guidance appears sufficient for effective ICB in children. However, prospective studies are needed.

## Figures and Tables

**Table 1:** Demographic data.

		US	US + NS	*p*
**Age**				0.774
Mean ± SD		9.29 ± 3.41	9.16 ± 3.42	
Min–Max (Median)		2–15 (9.5)	2–15 (9.5)	
95% CI		8.68, 9.90	8.55, 9.77	
**Gender**	Male n (%)	82 (68.3)	81 (67.5)	0.890
	Female n (%)	38 (31.7)	39 (32.5)	
**ASA**	No Comorbidity n (%)	115 (95.8)	117 (97.5)	0.722
	Comorbidity n (%)	5 (4.2)	3 (2.5)	
**Height**				0.917
Mean ± SD		137.3 ± 20.5	136.4 ± 20.4	
Min–Max (Median)		85–177 (138)	86–172 (139)	
95% CI		133.63, 140.97	132.75, 140.05	
**Weight**				0.774
Mean ± SD		36.2 ± 14.6	36.5 ± 14.5	
Min–Max (Median)		10–63 (33.5)	10–60 (36)	
95% CI		33.59, 38.81	33.91, 39.09	
**BMI**				0.306
Mean ± SD		18.1 ± 2.8	18,5 ± 3.0	
Min–Max (Median)		12.7–25.1 (18)	12.9–24 (18.4)	
95% CI		17.60, 18.60	17.96, 19.04	

US: ultrasound; US + NS: ultrasound with nerve stimulator; ASA: American Society of Anesthesia; BMI: body mass index; CI: Confidence Interval.

**Table 2:** Variation in some parameters between US and US + NS groups.

		US	US + NS	*p*
**Surgical Procedure Area**	Upper Arm (%)	35 (29.2)	36 (30.0)	0.769
	Forearm n (%)	65 (54.2)	68 (56.7)	
	Hand n (%)	20 (16.6)	16 (13.3)	
**Sedation**	Light (midazolam only) n (%)	72 (60.6)	68 (56.7)	0.784
	Deep (midazolam + ketamine) n (%)	48 (39.4)	52 (43.3)	
**Processing Time (min)**				<0.001 *
Mean ± SD		6.1 ± 0.8	8.31 ± 0.82	
Min–Max (Median)		5–8 (6)	7–10 (8)	
95% CI		5.96, 6.24	8.16, 8.46	
**Surgery Time (min)**				0.732
Mean ± SD		62.4 ± 11.3	62.4 ± 9.5	
Min–Max (Median)		30–82 (66)	34–76 (66)	
95% CI		60.38, 64.42	60.70, 64.10	

US: ultrasound; US + NS: ultrasound with nerve stimulator; min: minutes, * *p* < 0.05.

**Table 3:** Numerical comparisons of the pain scores applied to the groups.

	US	US + NS	*p*
**FLACC** n	42	44	
Mean ± SD	2.36 ± 0.87	2.30 ± 0.85	0.892
Min–Max (Median)	2–7 (3)	2–7 (3)	0.837
95% CI	2.22, 2.46	2.23, 2.51	
**VAS** n	78	76	
Mean ± SD	2.21 ± 0.83	2.18 ± 0.76	0.892
Min–Max (Median)	1–7 (3)	1–7 (3)	0.959
95% CI	2.11, 2.32	2.09, 2.27	

FLACC: Facial, Leg Movement, Crying, Availing Scale; VAS: Visual Analogue Scale; US: ultrasound; US + NS: ultrasound with nerve stimulator; n: sample size; CI: Confidence Interval.

**Table 4:** Comparison of postoperative FLACC scores of the groups.

	US	US + NS	*p*
**FLACC 1st hr**			0.559
Mean ± SD	1.10 ± 0.30	1.14 ± 0.35	
Min–Max (Median)	1–2 (1)	1–2 (1)	
95% CI	1.05, 1.15	1.08, 1.20	
**FLACC 3rd hr**			0.845
Mean ± SD	1.17 ± 0.44	1.18 ± 0.45	
Min–Max (Median)	1–3 (1)	1–3 (1)	
95% CI	1.09, 1.25	1.10, 1.26	
**FLACC 6th hr**			0.958
Mean ± SD	1.43 ± 0.94	1.45 ± 1.00	
Min–Max (Median)	1–5 (1)	1–5 (1)	
95% CI	1.26, 1.60	1.27, 1.63	
**FLACC 9th hr**			0.704
Mean ± SD	3.12 ± 1.19	2.98 ± 1.07	
Min–Max (Median)	1–6 (3)	1–5 (3)	
95% CI	2.91, 3.33	2.79, 3.17	
**FLACC 12th hr**			0.467
Mean ± SD	3.17 ± 1.29	3.32 ± 1.20	
Min–Max (Median)	1–7 (3)	2–7 (3)	
95% CI	2.94, 3.40	3.11, 3.53	
**FLACC 15th hr**			0.469
Mean ± SD	3.33 ± 1.28	3.55 ± 1.30	
Min–Max (Median)	1–6 (3)	2–7 (3)	
95% CI	3.10, 3.56	3.32, 3.78	
**FLACC 24th hr**			1.000
Mean ± SD	2.52 ± 0.67	2.48 ± 0.59	
Min–Max (Median)	2–5 (2)	1–4 (2)	
95% CI	2.40, 2.64	2.37, 2.59	

FLACC: Facial, Leg Movement, Crying, Availing Scale; US: ultrasound; US + NS: ultrasound with nerve stimulator; hr: hour, CI: Confidence Interval.

**Table 5:** Comparison of postoperative VAS of the groups.

	US	US + NS	*p*
**VAS 1st hr**			0.375
Mean ± SD	1.09 ± 0.29	1.05 ± 0.22	
Min–Max (Median)	1–2 (1)	1–2 (1)	
95% CI	1.04, 1.14	1.01, 1.09	
**VAS 3rd hr**			0.830
Mean ± SD	1.10 ± 0.31	1.09 ± 0.29	
Min–Max (Median)	1–2 (1)	1–2 (1)	
95% CI	1.04, 1.16	1.04, 1.14	
**VAS 6th hr**			0.560
Mean ± SD	1.35 ± 0.77	1.20 ± 0.43	
Min–Max (Median)	1–4 (1)	1–3 (1)	
95% CI	1.21, 1.49	1.12, 1.28	
**VAS 9th hr**			0.569
Mean ± SD	2.99 ± 1.26	3.09 ± 1.20	
Min–Max (Median)	1–6 (3)	1–6 (3)	
95% CI	2.76, 3.22	2.88, 3.30	
**VAS 12th hr**			0.713
Mean ± SD	3.18 ± 1.29	3.25 ± 1.28	
Min–Max (Median)	1–7 (3)	2–7 (3)	
95% CI	2.95, 3.41	3.02, 3.48	
**VAS 15th hr**			0.861
Mean ± SD	3.15 ± 1.21	3.14 ± 1.15	
Min–Max (Median)	1–5 (3)	1–5 (3)	
95% CI	2.93, 3.37	2.93, 3.35	
**VAS 24th hr**			0.176
Mean ± SD	2.64 ± 0.66	2.50 ± 0.60	
Min–Max (Median)	1–5 (3)	1–5 (2)	
95% CI	2.52, 2.76	2.39, 2.61	

VAS: Visual Analogue Scale; US: ultrasound; US + NS: ultrasound with nerve stimulator; hr: hour; CI: Confidence Interval.

**Table 6:** Comparison between groups in terms of block duration and analgesia.

		US	US + NS	*p*
**MBD**				0.460
Mean ± SD		6.20 ± 0.95	6.29 ± 0.88	
Min–Max (Median)		3–8 (6)	3–8 (6)	
95% CI		6.03, 6.37	6.13, 6.45	
**SBD**				0.381
Mean ± SD		9.38 ± 2.13	9.53 ± 2.05	
Min–Max (Median)		5–15 (9)	5–16 (9)	
95% CI		9.00, 9.76	9.16, 9.90	
**Intraoperative and postoperative patients given additional opioids** n (%)		0 (0)	0 (0)	−1
**Intraoperative and postoperative patients without additional opioids** n (%)		120 (100)	120 (100)	1
**Post Operative Non-Opioid Analgesia**	not given n (%)	6 (5.0)	8 (6.7)	0.582
	given n (%)	114 (95.0)	112 (93.3)	
**Time of first postoperative analgesic administration**				0.100
Mean ± SD		9.38 ± 2.13	9.68 ± 2.06	
Min–Max (Median)		5–15 (9)	5–16 (9)	
95% CI		9.00, 9.76	9.31, 10.05	
**Total number of analgesics given in the first 24 h postoperatively**				0.819
Mean ± SD		1.89 ± 0.59	1.88 ± 0.60	
Min–Max (Median)		1–3 (2)	1–3 (2)	
95% CI		1.78, 2.00	1.77, 1.99	

US: ultrasound; US + NS: ultrasound with nerve stimulator; MBD: motor block duration; SBD: Sensory Block Duration; CI: Confidence Interval.

## Data Availability

The data presented in this study are available on request from the corresponding author. The data are not publicly available due to privacy or ethical restrictions.
